# Study of Risk Factors Affecting the Survival Rate of Emergency Victims with “Chest Pain” as Chief Complaint

**DOI:** 10.4103/0970-0218.58385

**Published:** 2009-10

**Authors:** Biranchi N Jena, Adibabu Kadithi

**Affiliations:** Department of Research and Analytics, Emergency Management and Research Institute, Devar Yamzal, Medchal Road, Secunderabad – 500 014, Andhra Pradesh, India

**Keywords:** Emergencies with chest pain as chief complaint, risk factors, pre-hospital care record, response time

## Abstract

**Research Question:**

What are the risk factors affecting the survival of emergency victims with chest pain as chief complaint.

**Objectives:**

1. To find out the relative risk of different risk factors. 2. To find out whether the association between survival rate and various sociodemographic variables are statistically significant or not.

**Study Design:**

Descriptive study.

**Setting:**

This study is based on the Pre-hospital care Records (PCR) of the Emergency Management and Research Institute (EMRI) from May 2007 to December 2007, in Andhra Pradesh.

**Participants:**

2020 emergency victims, with chest pain as the chief complaint, reported to EMRI from May to December 2007.

**Study Variables:**

Demographic characteristics of the victims, time and day of the incident, response time in handling the emergency, and so on.

**Statistical Analysis:**

Proportions, Chi-Square test, and Odds Ratio.

**Results:**

Of all the risk factors studied, gender (Male), age (65 +), and incident location (residence), proved to be the risk factors for the non-survival of the victims of medical emergencies, with chest pain as the chief complaint. It was also observed that there was a statistically significant association (*P* < 0.05) between age, gender, area (urban and rural), and occupation with the survival rate. The response time was significantly associated with the survival rate, only for critical cases. Survival rate increases to 33% with response time less than 15 minutes from less than 5% with the response time more than 15 minutes.

## Introduction

Coronary heart disease is a leading factor causing morbidity and mortality, both in the developing and developed countries around the world. Angina pectoris (Chest pain caused by insufficient blood supply to the heart) and acute myocardial infarction (Heart attack) are the two most common features of coronary heart diseases, also known as coronary artery disease. According to the World Health Organization (WHO) estimates, in 2004, 17.1 million people around the world died from cardiovascular disease and the number is expected to grow to 23.4 million in 2030.(1) Chest pain is the most common initial symptom in patients diagnosed with coronary artery disease. “Non-specific” chest pain was the fourth most common cause of emergency visits, which accounted for 1.6 million visits in 23 selected states, in 2005, according to the latest news and numbers from the Agency for Healthcare Research and Quality. About one-fifth of the cases were admitted to hospitals for observation or treatment.(2) Out of the total number of patients who approached the Emergency Department with chest pain as the chief complaint, in the US, just five to 15 percent of them were found to be suffering from heart attacks or other cardiac diseases.(3) In countries like India, a sizable number of people seek emergency services with “Chest Pain” as the chief complaint. Therefore, it would be important to study the risk factors affecting the survival rate for those patients who sought the emergency services with chest pain as the chief complaint.

## Materials and Methods

The study is based on all the emergencies with chest pain as chief complaint, who reported to the Emergency Management and Research Institute (EMRI) from May 2007 to December 2007, in Andhra Pradesh, India. EMRI, born in 2005, in the state of Andhra Pradesh, provides emergency medical services along with Pre-Hospital Care. Pre-Hospital Care Record (PCR) was the major source of data for the study. PCR was introduced along with the operation of the Emergency Management and Research Institute (EMRI), providing pre-hospital care while transporting the emergency patients to the appropriate definite care units. PCR is an instrument that captures the socioeconomic–demographic variables of the emergency victims along with the pre-existing ailments and the type of medical intervention given to the victims during the pre-hospital care. As the PCR forms are filled by qualified and trained Emergency Medical Technicians (EMTs), the quality of data is assumed to be good for research projects. Therefore, the PCR is considered to be a good data source for studying the risk factors affecting the survival rate of emergency victims, with chest pain as chief complaint.

The study preliminarily scanned all the PCRs (7516) for emergencies who reported to the EMRI with a chief complaint of “chest pain”. However, based on the availability of variables identified for the study and the availability of the “Survival Status” of the victims, 2020 cases were included for the study. For the purpose of the study, the survival status was defined as whether the emergency victim was alive or not after 48 hours of providing the emergency transport with pre-hospital care to the patients. EMRI has a robust process for collecting the survival status of the victims after 48 hours of the incident. EMRI collects the information on the survival status in four major categories, namely, “alright and discharged from hospital”, “Stable, out of danger but still in the hospital”, “critical and still in the hospital,” and “expired”. In the current study, all the cases except “expired”, in the survival status, are considered to be “alive”.

The study is a descriptive study and uses the chi-square test for the significance in the association, odds ratio for the risk factors.

## Results

Out of the 2020 victims analyzed for chest pain, 92.23% (1863) had survived and 7.77% (157) had expired as per the “after 48 hours patient follow-up” information. It has been observed that more cases had been registered from the Krishna district (9.06%), followed by Chitoor (7.34%), and Guntur (7.33%). In the overall 2020, more cases of emergency with chest pain were recorded for males (58.16%) when compared to females (41.39%). The survival status shows that 9% of the male victims expired within 48 hours of the incident as compared to only 6% of the female victims. This indicates that the female were less susceptible to death resulting from “chest pain” as chief complaint as compared to their male counterparts, in the study of the selected reported cases to the EMRI. Age-wise analysis of victims revealed that a greater number of cases were reported for the people in the age group of 35 – 50 (32%) years. The mean age of the male victims was recorded as 49 years, whereas, the mean age for the female victims was comparatively low at 45 years. The mean age of the victims varied significantly (*P* < 0.001), with a mean age of 50 and 46 in the urban and rural areas, respectively. However, if we look at the total number of cases being reported from the urban and rural areas, it was observed that a greater number of cases were reported from the rural areas (83%) as compared to the urban areas (17%).

As EMRI has been providing emergency services across the state of Andhra Pradesh for over the last three years, the variation in reporting from the rural and urban areas could be due to better transport facility and proximity to health care facilities in the urban areas. The survival rate for the victims from rural areas is comparatively high at 93% as compared to the victims from urban areas (89%). The statistically significant difference (*P* < 0.05) shows that an emergency with chest pain as a chief complaint from the urban area is more likely to end in the non-surviving when compared with the reported cases from rural areas.

This study also suggests that reporting of such emergency is highest among the backward caste (38.27%) as compared to the general caste and Schedule Caste (SC)/Schedule Tribe (ST). There is not much variation in the survival rate among castes. A greater number of such emergencies occurred at residence (71.39%), than at the workplace (2.77%) or public place (16.14%). However the survival rate of such emergency occurrences in a public place is quite low (90%) as compared to the work place (97%).

As far as professional/occupational distribution of incidence is concerned, emergency cases are registered in daily wage workers (34.50%) followed by housewives (22.13%). However, the death rate is comparatively high in employees at 9.62%. Here too, work stress and psychological risk factors may influence the survival rate.

There is a variation among week days, as far as the number of cases being reported for medical emergency services. The number of cases reported was highest on Monday (18%) followed by Sunday (16%). However, there is no variation in the survival rate for such emergency cases reported on different days of the week.

A chi square test is run to show the association between different sociodemographic variables and the survival rate for the emergencies with chest pain as a major complaint. The result shows that variables like age and occupation are significantly associated with the survival status of the victim at 99% Confidence Interval (CI), whereas, variables like gender and area have an association with survival status of victim at 95% CI. However, variables such as caste, incident location, weekdays, and blood pressure, do not show any significant association with the survival of the victim of an emergency with chief complaint as chest pain [Table 1].

**Table 1:** *P* value of different variables showing the association with survival status

Variable	Chi-square test (*P* value)
Gender	0.02*
Age	0.00**
Area (Urban/Rural)	0.02*
Caste	0.15
Occupation	0.00**
Incident location	0.18
Week day	0.37
Blood pressure	0.11
Response time#	0.18

* Significant at 95% confidence interval

** Significant at 99% confidence interval

# Response time is defined as the total time taken from receiving the call at the Emergency Response Center (ERC) to reach the victim's place (scene), with the life saving ambulance. The contingency table for the Chi-square test is done on the basis of whether the response time has been less or more than 15 minutes and has the victim survived or not?

To measure the level of risk (NON survival of the emergency victims with chest pain as chief complaint) associated with different factors, an odds ratio test was performed [Table 2].

**Table 2:** Odds ratio, attributable risk rate, and excess rate ratio for the risk factors

Risk factor	Reference	Odds ratio	Attributable risk rate	Excess rate ratio
Hypertension (Systolic BP > 150 mm Hg)	≤ 150 mm Hg systolic	0.79	−0.24	−0.19
Age (65 + years)	≤ 65	3.86	0.70	2.31
Area (Rural)	Urban	0.63	−0.53	−0.35
Occupation (Daily wage worker)	Rest of the occupation	0.62	−0.55	−0.36
Gender (Male)	Female	1.52	0.32	0.47
Week Day (Monday)	Remaining weekdays	0.70	−0.40	−0.29
Time (7 pm to 11 pm)	Remaining hours	0.77	−0.28	−0.22
Response Time (more than 15 minutes)	≤ 15 minutes	0.80	−0.27	−0.20
Incident location (Residence)	Other places	1.03	0.029	0.029

The odds ratio for different risk factors revealed that age, gender, and incident location have the relative risk of non-survival in the event of the occurrence of emergency with chest pain as chief complaint. As compared to victims below 65 years, victims above 65 are four times more likely not to survive from the event of emergency with chest pain as chief complaint. Compared to females, males have 1.52 times greater risk of death in such emergencies. Incidence location does not have a significant relative risk; the occurrence of such an emergency at the residence exhibits a marginal risk. The systolic blood pressure with measurement of more than 150 mm Hg does not exhibit any risk for those victims reporting with emergencies of chest pain as chief complaint [Table 2].

Interestingly, the response time has not proved to be a risk factor for non-survival of emergency victims with chest pain. This is due to the fact that all the emergencies with chest pain as chief complaint are not life-threatening. Studies show that only five to 15% cases of chest pain cases turn out to be cardiac-related emergencies, which are life-threatening. As the diagnoses of chest pain cases are not captured in the present study, the mapping of life-threatening cases (which require quick pre-hospital care with less response time for better survival rate) with response time could not be done. The study further screens out those cases where Cardio Pulmonary Resuscitation (CPR) has been carried out by EMTs, to ascertain that such emergency cases are more likely cardiac-related emergencies. These cases are then mapped with the response time.

Out of 43 emergency cases with chest pain as chief complaint, where CPR was conducted by the EMT, the survival rate was significantly low (18.6%). However, if we look at the survival rate by the response time, it shows that the chance of survival increases significantly with better response time, with no further improvement in the pre-hospital care [Table 3]. It is evident from the analysis that the survival rate increases from 4.55% with the response time more than 15 minutes to 33.33% with response time less than 15 minutes.

**Table 3:** Survival status of victims by response time

Response Time	Saved	Expired	Total
< 15 min	7 (33.33)	14 (66.67)	11 (100.00)
≥ 15 min	1 (4.55)	21 (95.55)	22 (100.00)
Total	8 (18.60)	35 (81.40)	43 (100.00)

Figure in parentheses are not percentage

## Discussion

Extensive clinical and statistical studies have shown that several factors increase the risk and occurrence of cardiovascular disease. Among 1973 Emergency Department patients admitted with chest pain, 230 were given a diagnosis of chest pain of undetermined origin. Among patients discharged from the hospital with a diagnosis of chest pain of undetermined origin, those with an initial abnormal ECG, pre-existing diabetes, or pre-existing coronary artery disease are at higher risk of a subsequent adverse cardiac event. In the absence of such factors, the cardiac outcome is excellent.(4) Based on the study, it is deducted that around 11.7% of the total number of chest pain victims are diagnosed with some pre-existing cardiac-related ailments, and have a higher risk of death. Out of the total number of patients who approach the Emergency Department with a chief complaint of chest pain, in the US, just five to 15 percent of them are found to be suffering from heart attacks or other cardiac diseases(3) Proper evaluation of the patient with acute chest pain is a resource-intensive and expensive process. Physicians face enormous challenges in the management of these patients. The two major acute coronary syndromes (ACSs) that are encountered are acute myocardial infarction (AMI) and unstable angina pectoris. However, most chest pain patients do not have an ACS,(5) and most patients who do have an ACS have not had an AMI.(6) Therefore, most of the emergency victims seeking medical help because of chest pain, are less likely to have a cardiac-related emergency.

Shorter response time was significantly associated with increased probability of receiving defibrillation and survival to discharge, among those defibrillated. Reducing the response time to eight minutes from 15 minutes, increased the predicted survival to 8% and reducing it to five minutes increased the survival to 10 – 11%.(7) As the experience of AP shows that the mean response time in handling the emergencies with chief complaint of chest pain during the study period was 18.8 minutes, the survival rate was studied as a binary variable for critical cases, where CPR was administered as pre-hospital care. The response time was studied as more than 15 minutes and less than 15 minutes, and it showed that the survival rate increased by more than 28% in absolute terms if the response time was less than 15 minutes. Rade B Vukamir, in his study found that response time affected the survival rate in cardiac emergencies. He studied 874 pre-hospital cardiac arrest patients treated by urban, suburban, and rural emergency medical services and proved that survival was improved with a decrease in the response time (for BLS 5.52 minutes versus 6.81 minutes, for ACLS 7.29 minutes versus 9.49 minutes).(8)

The American Heart Association, in a study on “Blood pressure experience and risk of cardiovascular disease in the elderly,” shows a relationship between BP and CVD. The study reveals that blood pressure > 160 mm Hg adds a small, but statistically significant increment in predicting the future cardiovascular disease in the elderly.(9) When the systolic blood pressure was studied as a risk factor (for NON survival) along with severe chest pain, a blood pressure count of 160 mm Hg or more was not exhibiting as a risk factor nor showing a statistically significant association with the survival rate. The gender difference in the incidence and prevalence of heart disease is well established and mortality for coronary heart disease is greater in men than in women.(10–15) The present study also finds a similar pattern in the reporting of emergencies with chest pain as a major complaint by males and females. The reporting of male victims (58%) is more than the female victims (42%) and as far as the survival rate is concerned, female victims have a better survival rate (94%) than their male counterparts (91%) [*P* < 0.05].

D.R Witte, D.E. Grobee from Julius Center for Health Science and Primary Care, University Medical Center Utrecht- Netherlands, performed a meta-analysis in cardiac emergency and concluded that excess cardiac mortality occurs on Monday.(16) In the current study also it is evident that the highest number of such emergencies were registered on Monday (17.7%), however, as far as survival in an emergency with chest pain as chief complaint is concerned, Monday has not been proved to be a risk factor.

Chest pain with diabetes, for male victims of more than 70 years, is the intermediate likelihood for acute coronary syndrome.(17) The present study implies that male victims of more than 65 years are more prone to death with the onset of the medical emergency with chest pain as chief complaint.

## Conclusion

Out of all the medical emergencies with chest pain as chief complaint reported to the EMRI, around 10% of the cases are expected to be cardiac-related emergencies. Of all the risk factors studied, gender (Male), age (65+), and incident location (residence) have proved to be the risk factors for non survival of the victim in such emergencies. It is also observed that there is a statistically significant association (*P* < 0.05) between age, gender, area (urban and rural), and occupation, with the survival rate. As all the emergency cases with chest pain as the major complaint are not life-threatening, the response time is neither significantly associated with the survival status, nor has it proved to be a risk factor. However, the response time studied for the critical cases has proved that it is associated with the survival rate of the emergency victims. It has been found that the survival rate increases to 33% with a response time of less than 15 minutes from 6% with the response time of more than 15 minutes.

Thus, by improving the response time especially for male victims with age more than 65 years, more lives can be saved.
